# The Cajal school and the physiological role of astrocytes: a way of thinking

**DOI:** 10.3389/fnana.2014.00033

**Published:** 2014-05-19

**Authors:** Marta Navarrete, Alfonso Araque

**Affiliations:** ^1^Functional and Systems Neurobiology, Instituto Cajal, CSICMadrid, Spain; ^2^Department of Neuroscience, University of MinnesotaMinneapolis, MN, USA

**Keywords:** astrocytes, neuron-glia communication, Cajal, tripartite synapses, gliotransmission

## Abstract

Cajal is widely recognized by the scientific community for his important contributions to our knowledge of the neuronal organization of the nervous system. His studies on neuroglial cells are less recognized, yet they are no less relevant to our current understanding of the cellular bases of brain structure. Two pioneering studies published a century ago –“Something about the physiological significance of neuroglia” (Ramón y Cajal, 1897) and “A contribution to the understanding of neuroglia in the human brain” (Ramón y Cajal, 1913)—focused on glial cells and their role in brain physiology. Novel findings obtained using state-of-the-art and sophisticated technologies largely confirm many of the groundbreaking hypotheses proposed by Cajal related to the structural-functional properties of neuroglia. Here we propose to the reader a journey guided by the ideas of Cajal through the recent findings on the functional significance of astrocytes, the most abundant neuroglial cell type in the nervous system. Astrocyte–neuron interaction, which represents an emerging field in current neuroscience with important implications for our understanding of the cellular processes underlying brain function, has its roots in many of the original concepts proposed by Cajal.

One hundred years ago Cajal published two studies centered on glial cells: “Algo sobre la significación functional de la neuroglia” (Something about the physiological significance of neuroglia) in 1897 and “Contribución al conocimiento de la neuroglia del cerebro humano” (A contribution to the understanding of neuroglia of the human brain) in 1913, which proposed pioneering concepts regarding the relevance of glial cells in brain function that in many instances have been confirmed by recent evidence obtained using novel and sophisticated techniques. In this article, we propose to the reader a voyage starting from Cajal's original ideas to the most recent evidence revealing the functional significance of the neuroglia. While we will see how he opened new venues for the understanding of neuroglia and how his ideas have been largely confirmed by later studies, he must not be considered a scientific visionary; rather, he had an unparalleled capacity to extract general and dynamic physiological conclusions from observations of static images (Figure 1).

**Figure 1:**
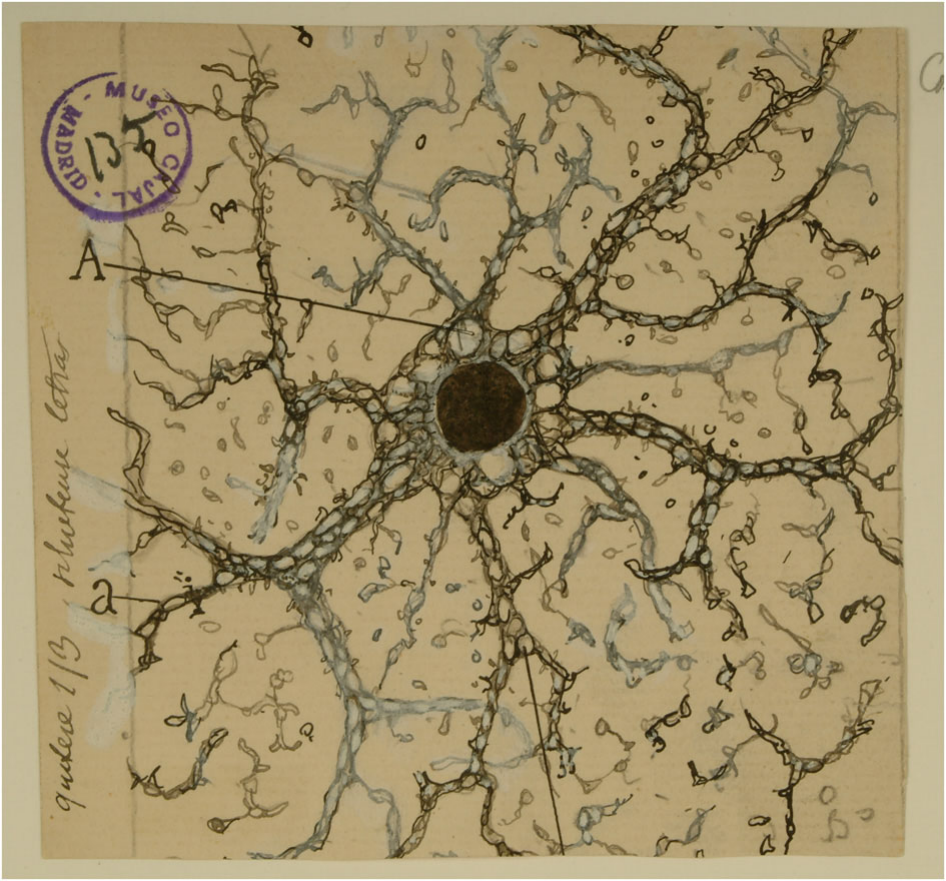
**Cajal's drawing showing a “neuroglia” of the pyramidal layer and stratum radiatum of the Ammon horn from adult man autopsied 3 h after death**. (A) Indicates the large vacuoles of the soma; (a and b), the gaps of the expansions intended for the gliosomas. Reproduced from an original drawing, with permission of the Instituto Cajal.

**¿“Qué significación funcional debemos otorgar a la neuroglía? Desgraciadamente, en el estado actual de la ciencia no es posible contestar a la importante pregunta más que mediante conjeturas más o menos racionales. En presencia de este problema, el fisiólogo se halla, por falta de métodos, totalmente desarmado”**

*What functional significance can be attributed to the neuroglia? Unfortunately, the present state of science does not allow to answer this important question but through more or less rational conjectures. When facing this problem, the physiologist is totally disarmed for lack of methods* (Ramón y Cajal, 1899).

This sentence reflects the incipient state of the technology at the end of the nineteenth century, which, however, did not prevent him from proposing ideas and hypotheses that were not fully misguided. At that time, one of the prevailing ideas concerning the function of glial cells postulated that they served to provide structural consistency to the nervous system in areas not occupied by neurons. In contrast, Cajal disagreed with this simple function ascribed to the neuroglia: ***¿Qué van a sostener corpúsculos pequeñísimos, aislados, flexibles, delicadísimos, mucho más delicados y pequeños que las células nerviosas mismas?*** (*What could hold these tiny, isolated, flexible, very delicate cells, much more delicate and smaller than the nerve cells?*) (Ramón y Cajal, 1895). In contrast, he proposed the *insulation theory*, that is, that astrocytes would serve as cellular insulators that separated the activity of neighboring neurons. While this hypothesis was not confirmed by subsequent studies, it is noteworthy that it was probably the first time that a direct involvement of astrocytes in neuronal function was proposed. Indeed, Cajal indicated “***No estimamos las hipótesis que acabamos de exponer como teorías exentas de reproche. Pero no por esto las hipótesis racionales, que tienen su punto de partida en algunos hechos conocidos, dejan de ser legítimas y hasta fecundas. Una hipótesis científica representa una dirección nueva, un camino que se traza a la observación y a la experimentación, el cual, si no conduce inmediatamente a la verdad, suscita siempre investigaciones y críticas que nos aproximan a ella***” (*We do not consider this hypothesis exempt from reproach. But rational hypotheses based on some actual facts are legitimate and even fruitful. A scientific hypothesis represents a new direction, a path traced for observation and experimentation, which, if it does not immediately lead to the truth, always raises investigations and criticisms that bring us closer to it*) (Ramón y Cajal, 1895).

In the 1980s, cellular biology and neuroscience underwent a technological revolution led by the development and use of novel tools such as the patch-clamp technique, fluorescence imaging, and confocal and multiphoton microscopy, which allowed the detailed visualization of structural properties and physiological processes of cells. Using these novel techniques, the physiologist was no longer unarmed but rather endowed with an arsenal of potent tools to investigate the function of astrocytes. Until then, astrocytes were considered to simply provide trophic and structural support for neurons. This *Nutrition Theory* originally proposed by Golgi (1903) has been consistently confirmed, and astrocytes are recognized as fundamental cells providing the necessary metabolic and nutritional support for the proper development and function of neurons (for a review see, e.g., Bélanger et al., 2011). However, this theory limited the function of astrocytes to a passive role without direct involvement in information processing in the brain. Nevertheless, recent evidence indicates astrocytic glycogen breakdown and lactate release is essential for the maintenance of long-term synaptic strength, suggesting that metabolic support from astrocytes is required for long-term memory formation (Suzuki et al., 2011).

The idea that astrocytes play passive roles in brain function probably derived from the fact that electricity is the main biophysical substrate underlying brain physiology. While neurons use electrical events to convey information, astrocytes are not electrically excitable cells. Furthermore, neurons were recognized to be directly in contact with the external world, receiving information from the sensory organs and transmitting information to endocrine organs and muscles. In contrast, astrocytes are confined to the central nervous system without direct physical communication with the environment. However, the use of fluorescence microscopy and calcium-sensitive fluorescent dyes in the decade of 1990s revealed that astrocytes display cellular excitability based not on electrical events but on variations in intracellular calcium concentration (Cornell-Bell et al., 1990; Charles et al., 1991; Perea and Araque, 2006; Perea et al., 2009; Zorec et al., 2012), which serve as cellular signals with important consequences for the physiology of the nervous system. Indeed, transient variations in cytosolic calcium in astrocytes occur spontaneously, but more importantly, they can also be evoked by synaptic activity and sensory stimuli, indicating that astrocytes sense neuronal activity and synaptic transmission (Wang et al., 2006; Perea et al., 2009; Takata et al., 2011; Navarrete et al., 2012; Araque et al., 2014). Indeed, astrocytes express several G protein-coupled neurotransmitter receptors, which upon stimulation activate phospholipase C leading to inositol triphosphate (IP_3_) production and calcium mobilization from internal stores. This astrocyte calcium signal can be elicited by a wide variety of neurotransmitters released from synaptic terminals, such as glutamate, gamma-aminobutyric acid (GABA), norepinephrine, dopamine, acetylcholine, serotonin, adenosine triphosphate (ATP) and nitric oxide (for reviews see Perea et al., 2009; Araque et al., 2014). Endocannabinoids released from postsynaptic neurons can also signal to astrocytes (Navarrete and Araque, 2008, 2010; Min and Nevian, 2012).

Consequently, astrocytes are now recognized to receive signals from neurons, actively responding to neuronal and synaptic activity with cytosolic calcium elevations evoked by neurotransmitters.

While synaptic activity is the input signal detected by astrocytes, what is the output of the astrocytic activity and what are its functional consequences?

***“La neuroglía de la substancia gris vendría a constituir una vasta glándula endocrina intercalada entre las neuronas y plexos nerviosos, destinada quizás a elaborar hormonas asociadas a la actividad cerebral”** (The gray matter neuroglia would constitute a vast endocrine gland intertwined with neurons and nerve plexus, intended perhaps to produce hormones associated with the brain activity)* (Ramón y Cajal, 1913).

Accumulating evidence obtained in the last 15 years has confirmed this original idea expressed by Cajal. Indeed, astrocytes can release neuroactive substances, called gliotransmitters, which include glutamate, GABA, ATP/adenosine, or D-serine (for reviews see Volterra and Meldolesi, 2005; Perea et al., 2009; Araque et al., 2014). These gliotransmitters may activate receptors in neurons, exerting different and complex effects depending on the neuronal receptor subtypes activated and their pre- or postsynaptic localization, that result in the regulation of the neuronal excitability and synaptic transmission and plasticity (Volterra and Meldolesi, 2005; Perea and Araque, 2010; Araque et al., 2014). Moreover, astrocyte signaling can regulate neural network function (Porto-Pazos et al., 2011), as recently described in the cortex where astrocytes regulate UP states (Poskanzer and Yuste, 2011; for a review see Araque and Navarrete, 2010) (Figure 2).

**Figure 2:**
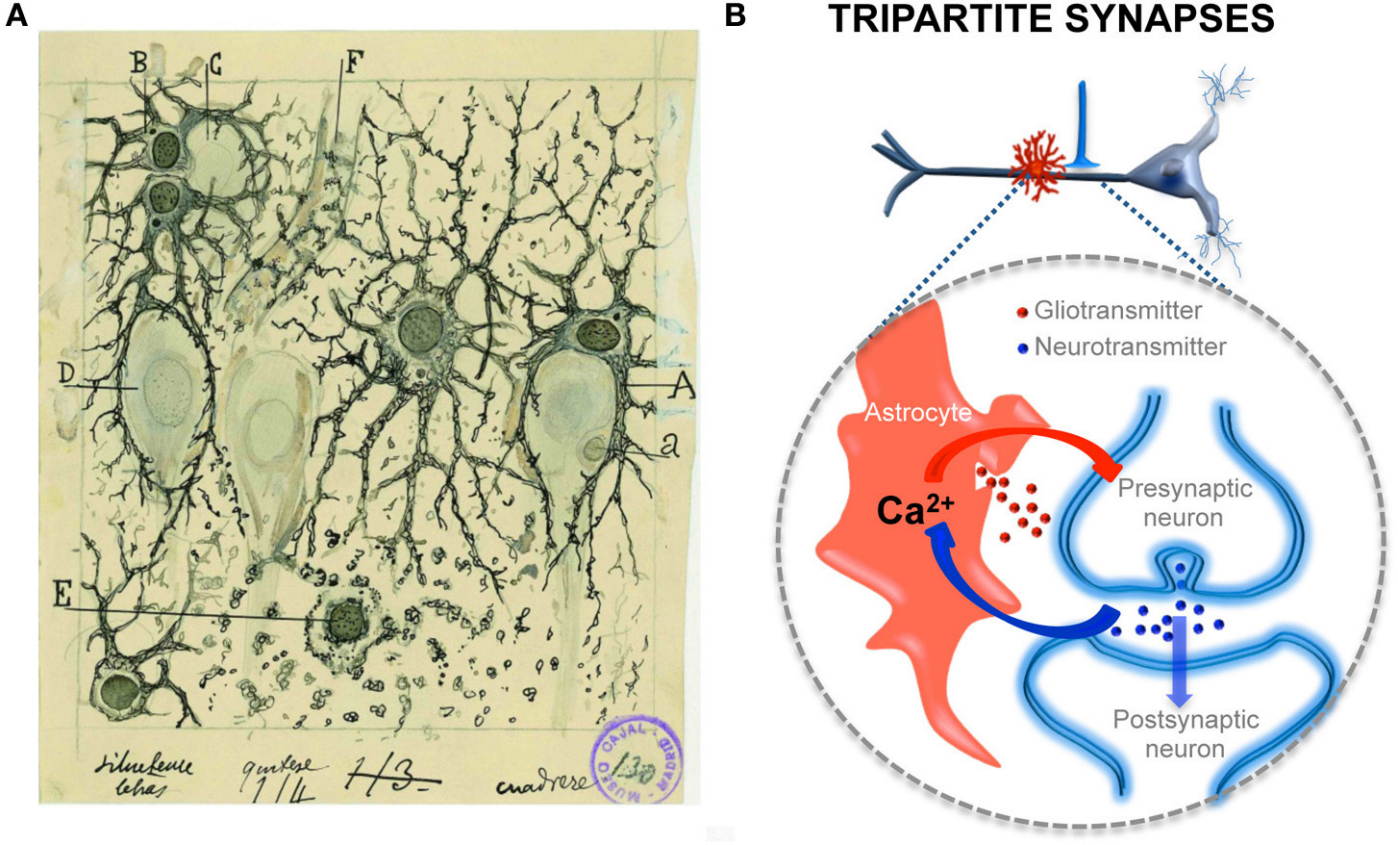
**Structural and functional relationships of neurons and astrocytes and tripartite synapses**. **(A)** Cajal's drawing showing “neuroglia” of the pyramidal layer and stratum radiatum of the Ammon horn (from adult man autopsied 3 h after death). Original labels: A, large astrocyte embracing a pyramidal neuron; B, twin astrocytes forming a nest around a cell, C, while one of them sends two branches forming another nest, D; E, cell with signs of “autolysis”; F, capillary vessel. Sublimated gold chloride method. Reproduced from an original drawing, with permission of the Instituto Cajal. **(B)** Scheme of one axon establishing a synapse on an apical dendrite of a prototypical pyramidal neuron and an astrocyte located close to it (in red). The large dashed circle illustrate an enlarged schematic view of the tripartite synapse, where the pre- and postsynaptic neuronal elements (in blue) are surrounded by astrocytic processes (in red). It also depicts the transfer of information between neuronal synaptic elements and astrocytic processes. Astrocytes respond with Ca^2+^ elevations to neurotransmitters (blue dots) released during synaptic activity and, in turn, control neuronal excitability and synaptic transmission through the Ca^2+^ -dependent release of gliotransmitters (red dots).

Evidence demonstrating the calcium-based astrocytic excitability elicited by synaptic activity and the calcium-dependent release of gliotransmitters that control synaptic transmission and plasticity has led to the establishment of a new concept in synaptic physiology, the Tripartite Synapse, in which astrocytes are integral elements of the synapses and actively exchange information with the neuronal elements (Araque et al., 1999; Halassa et al., 2009; Perea et al., 2009). This concept implies that the astrocytes directly play active roles in the transfer and storage of information in the brain and that the coordinated action of both neurons and astrocytes are involved in brain function.

Therefore, recent experimental findings regarding astrocyte physiology are in agreement with the original ideas expressed by Cajal based on observations of purely morphological data and on acute interpretation of those observations. Indeed, as he noted in 1897, “***Ciertos focos grises, ricos en plexos de expansiones dendríticas y de arborizaciones nerviosas, contienen muchas fibrillas de neuroglía y, al revés, ciertos focos pobres en dichos apéndices, son asimismo escasos en corpúsculos de Deiters o neuróglicos [astrocitos]***” and “***La neuroglía abunda donde las conexiones intercelulares son numerosas y complicadas, y no por el hecho de existir contactos, sino con la mira de reglarlos y dirigirlos de manera que cada expansión protoplásmica solo se ponga en relación íntima con un grupo especial de ramificaciones nerviosas terminales***” *(Certain gray nuclei enriched with plexus of dendritic expansions and nerve arborizations contain many neuroglia fibrils and, conversely, certain nuclei containing few of these appendices are also scarce in Deiters or neuroglia corpuscles [astrocytes]* and *the neuroglia is abundant where intercellular connections are numerous and complicated, not due to the existence of contacts, but rather to regulate and control them, in such a manner that each protoplasmic expansion is in an intimate relationship with only a particular group of nerve terminal branches)* (Ramón y Cajal, 1897).

Besides the intimate contact of astrocytes with synapses, they are also in close contact with blood vessels and capillaries. Cajal also proposed the physiological importance of astrocytes in the regulation of brain microcirculation.

**“Todo astrocito de la substancia blanca o gris está provisto de un aparato chupador o pedículo perivascular. El aparato chupador constituye no sólo una disposición constante de los astrocitos de la substancia blanca, sino uno de los factores neuróglicos más importantes de los centros. Semejante generalidad, junto con el hecho de que en los animales de pequeña talla (conejo, cobaya, etc.), y en los en curso de evolución (perro y gato de pocos días), el órgano chupador constituye la más espesa, y a veces la única expansión perceptible y bien coloreable del astrocito denotan que el susodicho apéndice debe desempeñar cometido fisiológico de primer orden.”**

*(Every astrocyte of the white or gray matter is provided with a sucking apparatus or perivascular pedicle [end foot]. The end foot is not only a constant characteristic of astrocytes in the white matter, but one of the most important neuroglial factors in the centers. Such generality, along with the fact that in small animals (rabbit, guinea pig, etc.) and in developing animals (few day-old cats and dogs) the end foot is the thickest, and sometimes the only perceptible and well-stained expansion of the astrocyte, indicates that such an appendage must play a first-order physiological role)* (Ramón y Cajal, 1913).

**“El objeto de tales elementos es suscitar, por contracción de los referidos apéndices, dilataciones locales de los vasos, y, por ende, congestiones fisiológicas ligadas a la mayor o menor intensidad de los procesos psíquicos.”**

*(The purpose of these elements is to provoke, by contraction of such appendages, local dilation of the vessels, and thus physiological congestions linked to the intensity of the mental processes)* (Ramón y Cajal, 1895).

Astrocytes are currently recognized as key elements involved in the regulation of brain capillary blood flow during functional hyperemia, that is, the local increase in blood flow produced during neuronal activity that allows the local delivery of oxygen and nutrients in functionally active brain regions with greater energetic requirements. Indeed, recent findings have shown that regional increases in astrocyte calcium levels, produced by neurotransmitter release during neuronal and synaptic activity, stimulate the release of gliotransmitters and vaso-active compounds that regulate localized dilation or constriction of brain capillaries (for recent reviews see Iadecola and Nedergaard, 2007; Carmignoto and Gomez-Gonzalo, 2010; Petzold and Murthy, 2011; Newman, 2013) (Figure 3), providing compelling evidence for Cajal's idea proposed more than a hundred years ago.

**Figure 3:**
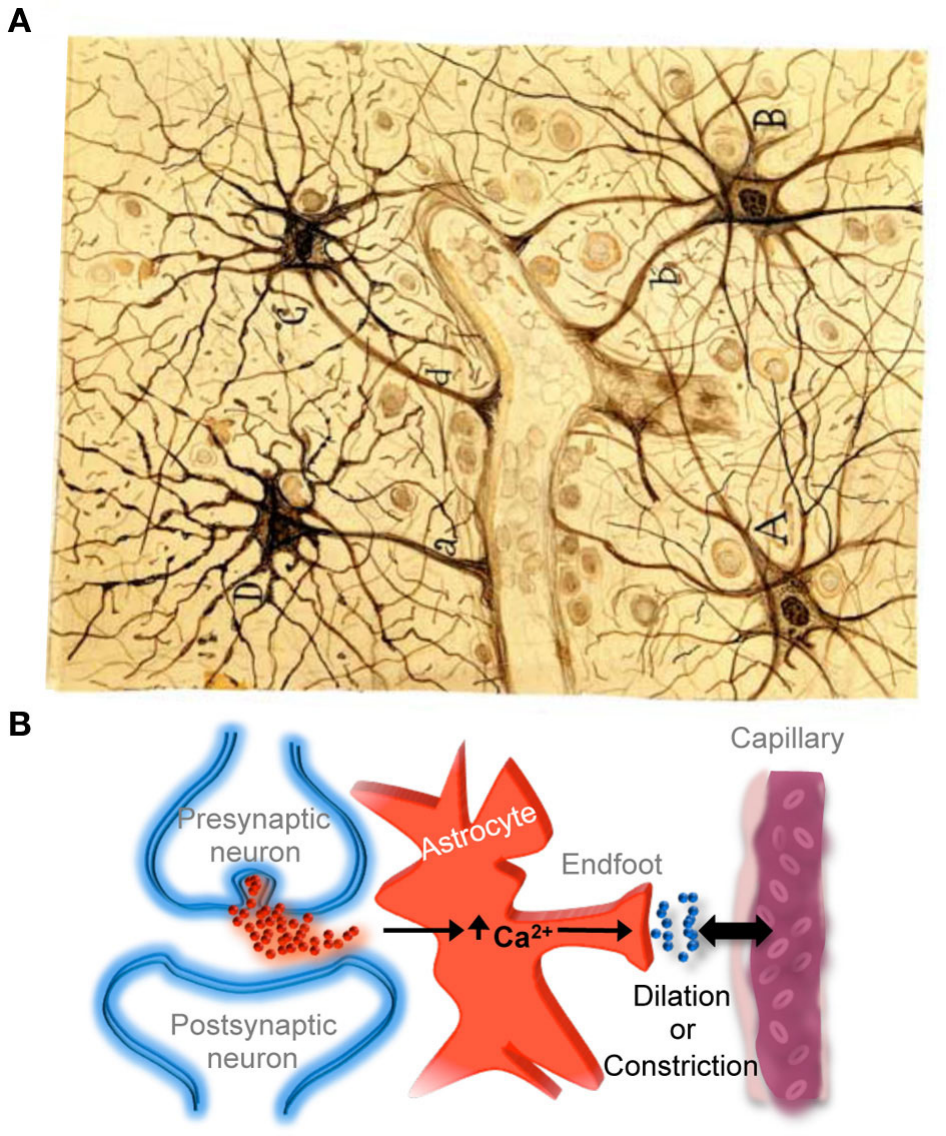
**Astrocyte-mediated control of neurovascular coupling**. **(A)** Cajal's drawing of fibrous astrocytes of human cerebral cortex surrounding a blood vessel. The original slide was impregnated by the sublimated gold chloride method. Reproduced from an original drawing, with permission of the Instituto Cajal. **(B)** Scheme illustrating that neuronal synaptic activity can signal astrocytes to regulate cerebral microcirculation. It should be noted that much of the neurovascular coupling is achieved through effects on smooth muscle cells that are present in arterioles but not in capillaries.

***“La corteza cerebral humana discrepa de la ·de los animales, no sólo por la cantidad enorme de células de tipo glandular que contiene, sino por la pequeñez de éstas y la riqueza del plexo gliomatoso intersticial.”** (The human brain cortex differs from that of other animals not only in the huge amount of glandular cells [astrocytes] that contains, but in their smallness and the wealth of the interstitial glial plexus)* (Ramón y Cajal, 1913).

This initial observation made by Cajal has also been confirmed by recent evidence showing that human neocortical astrocytes are larger and extend more primary processes than those of non-primate mammals (Oberheim et al., 2006, 2009; Matyash and Kettenmann, 2010). Moreover, some special anatomically defined subclasses of astrocytes are specifically present in the human neocortex (Oberheim et al., 2009). Based on this evidence, it has been proposed that astrocytic complexity has permitted the increased functional competence of the adult human brain (Oberheim et al., 2006, 2009; Navarrete et al., 2013). In addition and in agreement with Cajal's idea, it is noteworthy that the ratio between glial cells vs. neurons increases along the phylogenetic scale, e.g., from around 0.1 in nematodes to around 10 in primates (Sherwood et al., 2006; Herculano-Houzel, 2012; Lewitus et al., 2012). Although an exhaustive quantification is still lacking, the high number of glial cells in mammals with superior brain functions may be indicative that astrocytes may provide a greater degree of complexity and computational capacity to the brain. Likewise, it is noteworthy that during human evolution, the brain size increased around 300% with respect to its primitive ancestors, whereas the number of neurons only increased by 125% (De Felipe, 2011). Therefore, the major difference in brain volume between humans and primates is not only due to a higher development of the neuronal neuropil but also to a higher number and complexity of astrocytes. Perhaps what makes us human is in part due to astrocytes.

In conclusion, the naïve notion that the function of glial cells in general, and astrocytes in particular, was merely to provide trophic and structural support, with no relevant contribution to brain function, has been overcome by recent findings obtained using sophisticated experimental techniques. These findings demonstrate that astrocytes are active cellular players involved in the processing, transfer, and storage of information by the nervous system (Figure 2). In many cases, these findings have confirmed experimentally many of the original ideas proposed by Cajal regarding the physiological significance of neuroglia.

***“El prejuicio de que las fibrillas neuróglicas son a las células nerviosas lo que los haces colágenos del tejido conectivo a los corpúsculos musculares o glandulares, es decir, una trama pasiva de mero relleno y de sostén (y cuando más, una ganga destinada a embeberse en jugos nutritivos), constituye sin duda el principal obstáculo que el observador necesita remover para formarse un concepto racional de la actividad de los corpúsculos neuróglicos.”** (The prejudice that the relation between neuroglial fibers and neuronal cells is similar to the relation between connective tissue and muscle or gland cells, that is, a passive for merely filling and support (and in the best case, a gangue for taking nutritive juices), constitutes the main obstacle that the researcher needs to remove to get a rational concept about the activity of the neuroglia)* (Ramón y Cajal, 1899).

## Conflict of interest statement

The authors declare that the research was conducted in the absence of any commercial or financial relationships that could be construed as a potential conflict of interest.
